# Vaccinia Virus Shuffling: deVV5, a Novel Chimeric Poxvirus with Improved Oncolytic Potency

**DOI:** 10.3390/cancers10070231

**Published:** 2018-07-10

**Authors:** Marine Ricordel, Johann Foloppe, Delphine Antoine, Annie Findeli, Juliette Kempf, Pascale Cordier, Aude Gerbaud, Benoit Grellier, Monika Lusky, Eric Quemeneur, Philippe Erbs

**Affiliations:** Transgene SA, 400 Bld Gonthier d’Andernach, 67400 Illkirch-Graffenstaden, France; mricordel@polyplus-transfection.com (M.R.); foloppe@transgene.fr (J.F.); delphine.antoine@hotmail.fr (D.A.); findeli@transgene.fr (A.F.); kempf@transgene.fr (J.K.); pascale.cordier@transgene.fr (P.C.); gerbaud@transgene.fr (A.G.); grellier@transgene.fr (B.G.); lusky@transgene.fr (M.L.); quemeneur@transgene.fr (E.Q.)

**Keywords:** vaccinia virus, directed evolution, genome shuffling, deVV5-*fcu1*

## Abstract

Oncolytic virus (OV) therapy has emerged as a promising approach for cancer treatment with the potential to be less toxic and more efficient than classic cancer therapies. Various types of OVs in clinical development, including Vaccinia virus (VACV)-derived OVs, have shown good safety profiles, but limited therapeutic efficacy as monotherapy in some cancer models. Many different methods have been employed to improve the oncolytic potency of OVs. In this study, we used a directed evolution process, pooling different strains of VACV, including Copenhagen, Western Reserve and Wyeth strains and the attenuated modified vaccinia virus Ankara (MVA), to generate a new recombinant poxvirus with increased oncolytic properties. Through selective pressure, a chimeric VACV, deVV5, with increased cancer cell killing capacity and tumor selectivity in vitro was derived. The chimeric viral genome contains sequences of all parental strains. To further improve the tumor selectivity and anti-tumor activity of deVV5, we generated a thymidine kinase (TK)-deleted chimeric virus armed with the suicide gene *FCU1*. This TK-deleted virus, deVV5-*fcu1* replicated efficiently in human tumor cells, and was notably attenuated in normal primary cells. These studies demonstrate the potential of directed evolution as an efficient way to generate recombinant poxviruses with increased oncolytic potency, and with high therapeutic index to improve cancer therapy.

## 1. Introduction

The field of oncolytic virotherapy has gained considerable importance in the armamentarium of novel cancer therapies in recent years. In addition to their direct oncolytic activity oncolytic viruses (OVs) are increasingly recognized as an immunotherapy by inducing and amplifying host cell immune responses, leading to immunogenic cell death and long-lasting anti-tumor immunity [1,2,3]. Many different OVs have entered into clinical use. Imlygic^TM^ (talimogene laherparevec, also formerly known as T-VEC), an oncolytic herpes virus expressing granulocyte-macrophage colony-stimulating factor (GM-CSF), was the first OV to gain approval from the U.S. Food and Drug Administration, and European Medicines Agency in 2015, for the loco-regional treatment of melanoma lesions in the skin and lymph nodes [4]. Many different approaches have been employed to improve the oncolytic potency of OVs, including combination therapies with standard and emerging anticancer therapies [5]. Combination of OVs with immune checkpoint inhibitors (ICI) has also demonstrated synergy [6]. Alternative approaches to improve the efficacy of OVs include the arming of OVs with either therapeutic transgenes or immune stimulatory genes [7]. 

Vaccinia virus (VACV) has been the most commonly used poxvirus vector for cancer therapy due to its excellent lytic activity, the capacity to infect a wide range of tumors and the ability to engineer its genome [8]. Moreover, it was shown to display natural tumor tropism, selectively targeting tumors after systemic administration [9]. Several strains of VACV are currently evaluated in preclinical and clinical trials including Wyeth, Western Reserve, Copenhagen and Lister strains [10,11,12,13]. VACV derived OVs have shown good safety profiles but have demonstrated limited therapeutic efficacy as monotherapy. Therefore, new approaches to generate highly potent OVs are needed.

A first approach consists to adapt OVs by serial passages on tumor cells. Using this method, Yoo et al. have selected an improved oncolytic VACV by repeated selective replication in cancerous tissues [14]. A second approach, based on homologous recombination and using the high DNA homology between viruses of the same family, enables the generation of potent OVs. Homologous recombination is the basis for many widely used genetic techniques in virus research, including construction of recombinant vectors [15]. The feasibility of intermolecular recombination between different VACV strains to generate viable hybrids was reported some decades ago [16,17], and it was recently shown that this mechanism could induce multiple genetic exchanges even after one round of selection [18]. Directed evolution is based on extensive intermolecular recombination, and is a method to mimic, and accelerate the process of natural selection to evolve virus under controlled culture conditions. The method was employed to create novel AAV gene delivery vectors [19], was also used by Kuhn and colleagues to improve the therapeutic index of oncolytic adenoviruses [20]. By pooling different Adenovirus (Ad) serotypes, and by passing them on human tumor cell lines to promote recombination and selection of potent viral variants, they selected ColoAd1 for its rapid and potent replication in colon cancer cells [20]. Recently, they also isolated a new chimeric adenovirus, named OvAd1, on 3D ovarian cell culture [21]. Using the same homologous recombination approach, an improved OV was selected by pooling different orthopoxviruses [22]. Based on these different results, we hypothesized that it should be possible to create new recombinant VACV variants with improved oncolytic properties.

Our starting pool consisted of a mix of three replicative VACV strains, Copenhagen, Western Reserve, and Wyeth, and of the non-propagative Modified Vaccinia of Ankara (MVA) strain. The rationale for choosing these four strains includes the fact they all have been employed in the smallpox vaccination campaign demonstrating a high safety profile [23], and/or have been extensively studied as viral vectors in both preclinical and clinical studies. Pexa-Vec (pexastimogene devacirepvec, JX-594), the most advanced VACV oncolytic product is a Wyeth (WY) engineered virus to express GM-CSF [10]. Pexa-Vec has now entered a randomized controlled Phase 3 trial in advanced first-line hepato-carcinoma (HCC) with the standard of care, sorafenib (National Clinical Trial NCT02562755). The safety, systemic spread and anti-tumor activity of a Western Reserve (WR)-based VACV has been recently demonstrated in a Phase 1 trial [11]. We used the Copenhagen stain (COP) to engineer a TK-deleted VACV expressing the *FCU1* fusion suicide gene for targeted prodrug therapy [12]. It displayed a highly potent anti-tumor effect both in vitro and in vivo, and TG6002, a derivative of this virus containing a second deletion for ribonucleotide reductase [1], has entered into clinical development in recurrent glioblastoma patients (National Clinical Trial NCT03294486). Modified Vaccinia of Ankara (MVA) is a highly attenuated, non-replicative VACV strain, generated by passaging the VACV Ankara strain more than 570 times in primary chicken embryonic fibroblasts (CEF), thereby losing the ability to produce infectious progeny virus in almost all mammalian cell lines, including human cells [24]. After being used intensively in the smallpox vaccination campaign, MVA has been widely studied as vaccine vector for infectious diseases and cancer [23]. A MVA armed with the suicide gene *FCU1* (TG4023) [25] has been evaluated in a Phase I trial in primary or metastatic liver tumors [26] and a MVA armed with the tumor-associated antigen MUC1 (TG4010) has been studied in a randomized controlled Phase 2b trial in non-small cell lung cancer, in combination with chemotherapy [27]. 

Through this directed evolution process, we selected a VACV hybrid, named deVV5, with enhanced oncolytic properties in a series of human cancer cell lines representing many human solid tumor types. In addition, deVV5 was further modified by inserting the *FCU1* gene [28] into the TK locus, under the control of the strong VACV promoter p11k7.5. The recombinant deVV5-*fcu1* virus displayed further increased replicative and oncolytic activity on various human tumor cells. This study demonstrates that, through shuffling of various VACV strains, novel OVs can be generated with improved oncolytic properties in tumor cells and increased attenuation in normal cells.

## 2. Materials and Methods

### 2.1. Cell Culture and Viruses

Human colon cancer cell lines LoVo (CCL-229^TM^) and HCT 116 (CCL-247^TM^), human lung cancer cell line A549 (CCL-185^TM^), human hepatocarcinoma cell line Hep G2 (HB 8065^TM^), human glioblastoma cancer cell line U-87 MG (HTB-14^TM^), human gastric carcinoma cell line KATO III (HTB-103TM), human pancreatic cancer cell line MIA PaCa-2 (CRL-1420^TM^), human ovarian cancer cell line SK-OV-3 (HTB-77TM), human bladder cancer cell line UM-UC-3 (CRL-1749TM) and human osteosarcoma cell line 143B, a Thymidine Kinase (TK)-deficient cell line, (CRL-8304^TM^) were obtained from the American Type Culture Collection (ATCC, Rockville, MD, USA). Human esophagus cancer cell line OE19 (n°96071721) was obtained from European Collection of Cell Culture (ECACC). Human head and neck cancer cell line CAL33 was kindly provided by Dr. G. Milano (Centre Antoine-Lacassagne, Nice, France). All cell lines were grown in recommended media supplemented with 10% fetal calf serum (FCS). Fresh human hepatocytes were purchased from Biopredic International (Rennes, France) and maintained in the recommended hepatocyte medium provided by the supplier (Biopredic International).

Primary chicken embryo fibroblasts (CEF) were used for recombination, production and titration of viral vectors. CEF cells were prepared from chicken embryos obtained from fertilized eggs (Charles River SPAFAS) previously incubated 11 or 12 days at 37 °C in a humid atmosphere. Chicken embryos were dissected and treated with a 2.5% (*w*/*v*) solution of trypsin. CEF cells were maintained in Eagle-based Medium (MBE) supplemented with 5% fetal calf serum. Wild type vaccinia viruses Wyeth strain (WY, VR-1536^TM^) and Western Reserve strain (WR, VR-119^TM^) were obtained from ATCC. Wild type vaccinia virus Copenhagen strain (COP) used in the work described here comes from the Institut Mérieux (Marcy l’Etoile, France). MVA expressing the *eGFP* gene under the control of the p11k7.5 promoter (MVA-GFP) was constructed and characterized previously [25]. 

### 2.2. 3D Skin Model

The Phenion full-thickness (FT) skin model is a 3D tissue construct that simulates histological and physiological properties of human skin (surface 1.3 cm^2^). It was purchased from Henkel AG & Company KGaA (Düsseldorf, Germany). According to the supplier’s instructions, tissues were kept at 37 °C under 5% CO_2_ overnight and fresh, pre-warmed medium was added. Each Phenion FT skin model was infected with 1 × 10^5^ PFU of virus. Cultures were incubated for 7 days at 37 °C and medium was changed every 2 days. After 7 days of infection, medium and skins were frozen. Viral replication was quantified on CEF by plaque assay after sonication.

### 2.3. Directed Evolution for Selection of Chimeric Vaccinia Virus deVV5

Four viruses COP, WR, Wy and MVA were mixed and used to infect human LoVo cells (1.5 × 10^6^ cells/well in a 6 well-plate) and constituted the passage 1 (LP1). The mix used for the first passage consisted of 1.5 × 10^4^ PFU of COP, WY and WR corresponding to a Multiplicity of Infection (MOI) of 10^–2^ for each virus and 1.5 × 10^5^ PFU of MVA-GFP corresponding to a MOI 10^–1^. LP1 was then used in its entirety to infect T-75 tissue culture flask (T75). Supernatants from the second passage were then used in a 10-fold dilution series to infect confluent T75 plates of LoVo cells. Amplification and selection of viral progeny was done by 9 successive passages on LoVo cells by supernatant dilution. The infected T75 were observed for the first signs of cytopathic effect (CPE). To harvest the most potent viruses, cell culture supernatant was harvested from the flask infected with the most concentrated innocula in the 10-fold dilution series that did not show any sign of potent CPE. LP2 and LP3 were harvested after 24 h of infection. Next passages (LP4 to LP9) were harvested at 72 h post infection. LP9 served as crude lysate for a new round of selection on MIA PaCa-2 cells. Twelve passages were performed on MIA PaCa-2 cells as described above. Viral selection and amplification of the strongest chimera was undertaken by decimal dilutions.

### 2.4. Generation of deVV5-fcu1

deVV5-*fcu1* was generated by insertion of the *FCU1* gene into the deVV5 *TK* locus. For TK inactivation, successful recombination events were selected as described [12]. Briefly, CEF were infected with deVV5 at a MOI of 10^–2^ and incubated at 37 °C for 2 h, then transfected by electroporation using nucleofector^TM^ 2b device (Amaxa GmbH, Cologne, Germany) with 2 µg of a shuttle plasmid containing the *FCU1* gene under the control of the synthetic p11k7.5 promoter and surrounded by the flanking sequence of the vaccinia virus *TK* gene. The cells were then incubated for 48 h at 37 °C. Double recombination occurred between TK homologous regions in the shuttle plasmid and the wild-type virus, resulting in the insertion of the *FCU1* gene into the *TK* locus of deVV5. Recombinant TK-deleted virus deVV5-*fcu1* was selected after infection of the TK-deficient 143B cells in selection medium containing 5-bromo-2-deoxyuridine at final concentration of 150 mg/mL (Sigma, St Louis, MO, USA). Positive TK-plaques were submitted to additional plaque purification cycles in the same conditions. Insertion of the FCU1 sequence into the *TK* locus was confirmed by multiple PCRs and DNA sequencing.

### 2.5. In Vitro Cytotoxicity Assay

The lytic capacity was measured using the trypan blue exclusion method. Human tumor cells were transduced in suspension by respective chimeric viruses at the indicated MOI. A total of 3 × 10^5^ cells/well were plated in 6-well culture dishes in 2 mL of medium supplemented with 10% FCS. Cells were then cultured at 37 °C for 5 days and the viable cells were counted by trypan blue exclusion using a Vi-Cell Cell Counter (Beckman Coulter, Brea, CA, USA). All samples were analyzed in triplicate. Mock-infected cells served as negative control and established the 100% survival point for the given assay. 

### 2.6. In Vitro Virus Yield Assay

Growing Hep G2, A549 and OE19 tumor cells were seeded onto 6-well plates at 3 × 10^5^ cells/well. After 24 h in DMEM with 10% FCS, the cells were infected with individual virus at MOI of 10^–5^ and cells were incubated in fresh growth medium supplemented with 10% FCS until harvesting. Supernatants and cells collected 72 h post infection were submitted to a quick freeze-thaw cycle and sonication to release intracellular viral particles and viral progeny were quantified on CEF by plaque assay. All samples were analyzed in triplicate. To evaluate viral replication human primary cells, human primary hepatocytes were infected in 6-well plates (1 × 10^6^ cells/well) by COP, deVV5 and deVV5-*fcu1* at a MOI of 10^–4^ (100 PFU/well). Cells were incubated in fresh growth medium supplemented with 10% FCS until harvesting. At 48 h post infection, supernatant and cells were collected, freeze-thawed and sonicated and viral progeny was quantified on CEF by plaque assay. To evaluate viral replication on human skin tissue, human reconstituted Phenion skins were infected. The Phenion full-thickness (FT) skin model, a three-dimensional tissue construct that simulates histological and physiological properties of human skin, was purchased from Henkel AG&Co. KGaA (Düsseldorf, Germany). This organotypic epithelial raft culture model was maintained in tissue culture medium according to the manufacturer’s instructions. Each Phenion FT skin model was infected with 1 × 10^5^ PFU of COP, deVV5-*fcu1* and deVV5 by infusion (viruses were added directly into the medium). Cultures were incubated for 7 days at 37 °C and medium was changed twice a week. Viral replication both in medium and reconstituted skin was quantified on CEF by plaque assay after 2 cycles of sonication in PBS.

### 2.7. In Vitro Cell Sensitivity to 5-FC

Human HCT 116 tumor cells in suspension were infected by deVV5 and deVV5-*fcu1* viruses at an MOI of 10^–5^. A total of 3 × 10^5^ cells/well were plated in 6-well culture dishes in 2 mL of medium supplemented with 10% FCS. After 48 h of infection, cells were exposed to various concentrations of 5-FC ranging from 10^–7^ to 10^–3^ M. Three days later, cell viability was determined by trypan blue exclusion using a Vi-Cell cell counter. Results are expressed as percentage of viable cells, 100% corresponding to infected cells without 5-FC.

### 2.8. Cytosine Deaminase Enzymatic Assay

Cytosine deaminase (CDase) activity was quantified by measuring the amount of 5-FU released in the culture media. HCT 116 cells were infected with the different vectors at a MOI of 10^–5^ and plated in 6-well culture dish (1 × 10^6^ cells/well). After 48 h, 0.1 mM 5-FC was added to the culture medium. Every day for 4 days, 5-FC and 5-FU concentrations in the media were measured by HPLC. Fifty µL of media were quenched with 50 µL of acetonitrile. The samples were vortexed and centrifuged. The organic supernatant was evaporated to dryness and reconstituted in 50 µL of water and analyzed by HPLC using a mobile phase of 50 mM phosphoric acid adjusted to pH 2.1. Results are expressed as the percentage of 5-FU relative to the total amount of 5FC + 5FU after various incubation times with 5-FC.

### 2.9. DNA Sequencing

Samples from viral DNA were purified with AMPure XP (Beckman Coulter Inc., Brea, CA, USA) beads kit to remove residual cellular DNA and sent for sequencing to the GenomEast platform (IGBMC Microarray and Sequencing platform, Illkirch-Graffenstaden, France). Data from Illumina HiSeq4000 (Illumina Inc., San Diego, CA, USA) in 2 × 100bp paired-end runs were quality trimmed using a Phred score threshold of Q30. Contigs were de novo assembled with SPAdes v3.11.1 [29] following authors instructions and scaffolding was performed with custom script to generate the longest consensus sequence for each viral genome. BLAST 2.7.1 [30] pairwise alignments were used to locate homologous regions between deVV5 and each of the parental strains. Global pairwise alignments were performed using MAFFT v7.017 [31] to highlight the longest regions with strict identity between deVV5 and the corresponding parental genome. Results were reported using Circos v0.69-3 [32] and Geneious v8.1.9, www.geneious.com software [33].

### 2.10. Statistical Analyses

Two-tailed Student’s was used to determine significance between treatment groups. Differences were considered significant at *p* < 0.05. 

## 3. Results

### 3.1. Oncolytic Activity of the Four VACV Parental Strains 

The oncolytic activity of the four parental VACV strains on various human cell lines was measured to serve as a reference (Table 1). The percentage of surviving cells was calculated 5 days post infection at a MOI of 10^–5^ (1 infectious viral particle for 10^5^ cells), with 100% of cell survival corresponding to the mock-infected cells. At this MOI, COP was the strongest oncolytic strain tested, therefore it was used as the reference for oncolytic potency throughout this study. As expected and as previously demonstrated [25], MVA showed no cytotoxicity on tumor cell lines. Table 1 also shows that the tumor cell lines LoVo and MIA PaCa-2 are among the most resistant to all three OVs: WY, WR and COP.

### 3.2. VACV Shuffling: deVV5 Is a Chimeric Virus with Enhanced Oncolytic Potency In Vitro 

**Figure 1:**
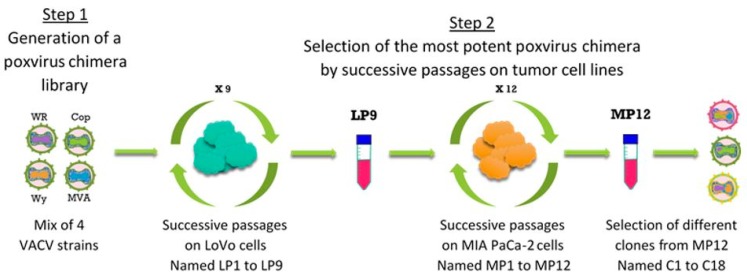
The directed evolution process. Representation of the different steps of the directed evolution process used to select a new Poxvirus chimera with improved oncolytic activity.

A directed evolution strategy employed to generate chimeric VACV comprised two steps (Figure 1). First, a library of viruses was generated by co-infecting LoVo cells with the four viral strains including COP, WY, WR and MVA. Second, amplification of viral progeny under stringent conditions towards clonal isolation of virus candidates was performed by nine successive passages on LoVo cells (LP1–LP9) followed by 12 successive passages on MIA PaCa-2 cells (MP1–MP12), both known to be rather resistant to OV compared to other tumor cell lines (see also Table 1). 

**Figure 2:**
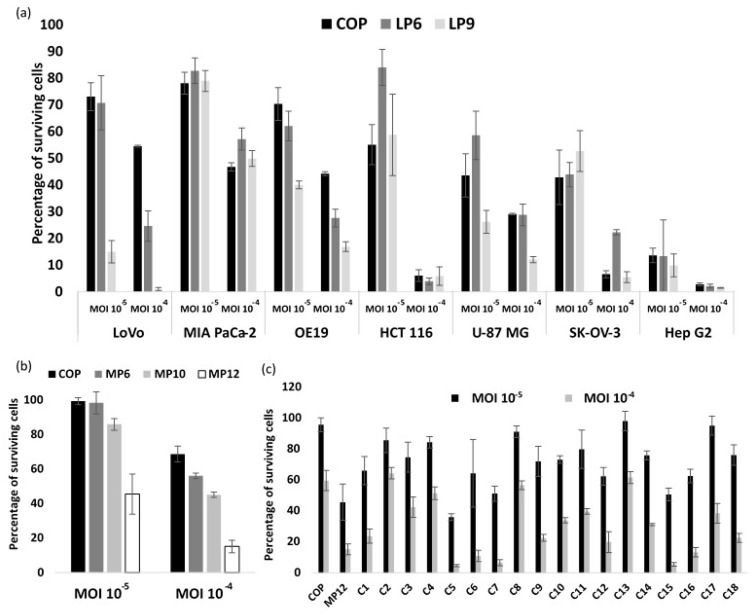
Oncolytic activities during the different steps of the directed evolution process. (**a**) Percentage of surviving cells 5 days after infection with passage 6 (LP6), passage 9 (LP9) and Copenhagen stain (COP). Human tumor cells were infected at a MOI of 10^–5^ and 10^–4^ with COP and the indicated passages and cell viability was determined by trypan blue exclusion. The parental COP was used as reference. The results are presented as a mean of triplicate experiments ± SD. (**b**) Percentage of surviving cells after infection of MIA PaCa-2 tumor cell line with the indicated passages at MOI 10^–5^ and 10^–4^. Cell viability was determined 5 days after infection. The parental COP was used as reference. The results are presented as a mean of triplicate experiments ± SD. (**c**) Percentage of surviving cells 5 days after infection of MIA PaCa-2 tumor cell line with the indicated clones at MOI 10^–4^ and 10^–5^. The parental COP and MP12 were used as references. The results are presented as a mean of triplicate experiments ± SD.

During the whole process of chimeric virus selection, several passages were performed by dilution imposing stringent selective pressure towards the genetic selection of a rare recombination event. Oncolytic activity of different passages (LP6 and LP9) were then tested in comparison to the parental COP (Figure 2a). At passage 6 (LP6), no improvement in cytopathic activity was observed except at MOI 10^–4^ in LoVo cells. At passage 9 (LP9), a clear gain of oncolytic potency was recorded on LoVo cells and a slight gain was observed in OE19 and U-87 MG cells. In contrast, no improvement of oncolytic activity was observed in SK-OV-3, HCT 116, Hep G2 and MIA PaCa-2 cells. Considering these results, LP9 was chosen for another cycle of genetic selection on MIA PaCa-2 which was the least permissive cell line. This second round of selection was performed in order to select an improved oncolytic virus in a wide range of tumor types. Selection by dilution was performed under the same conditions, as described above and in the Materials and Methods section. During the selection process in the MIA PaCa-2 cell line, three passages were tested for their lytic potential. MP6, MP10 and MP12 were compared at low MOI, with the parental COP (Figure 2b). MP12 was found to be more effective than all others. Based on these results MP12 was selected to proceed to clone isolation. Eighteen individual plaque-purified viruses were isolated and screened for their oncolytic potential on the MIA PaCa-2 tumor cell line. The oncolytic potency of individual plaque clones was compared to that of the parental COP and the pool MP12 (Figure 2c). The clones were found heterogenic in potency compared to MP12 pool, suggesting that numerous viruses were present in the pool. About half of the clones seemed to have a similar activity as MP12 (clones 1, 5, 6, 7, 9, 12, 15, 16 and 18) and the other half demonstrated an oncolytic activity close to that observed with COP. The most potent clone at both MOI 10^–5^ and 10^–4^ was clone C5. With this clone and using a MOI of 10^–5^, we have obtained a superior oncolytic activity compared to COP used at MOI 10^–4^. The clone purity was assessed by evaluation of 20 plaque-purified clones originated from C5, all of which displayed the same phenotype in different tumor cells (data not shown). This clone, termed deVV5, was chosen for further characterizations.

### 3.3. Genome Analysis

**Figure 3:**
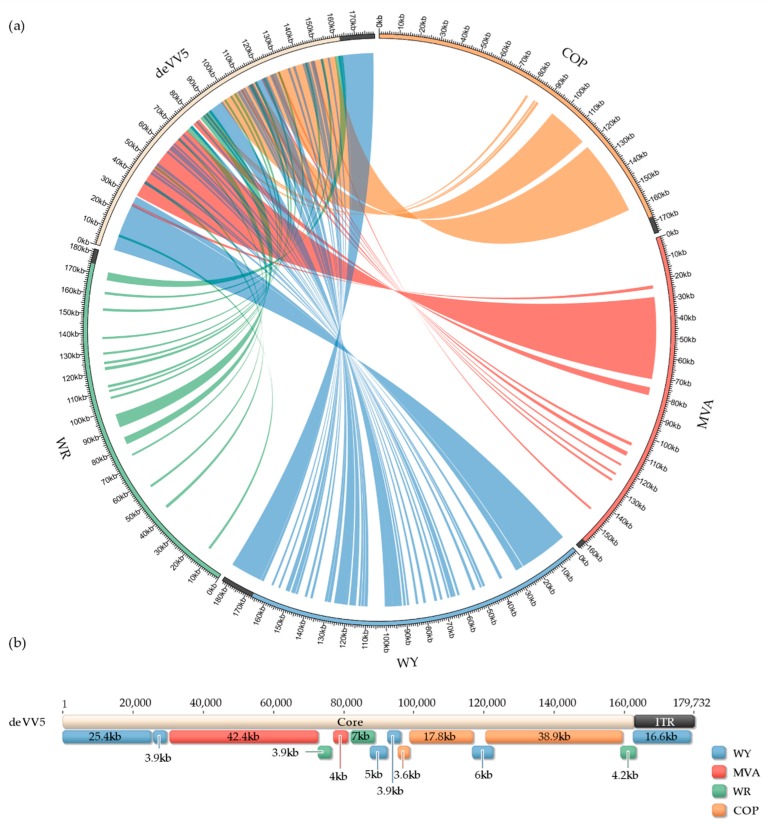
Homology maps between deVV5 and each parental genome. (**a**) De novo sequenced viral genomes are shown around the circos plot (COP, orange; MVA, red; WY, blue; WR, green; deVV5, white). Only one ITR is displayed (black). Blast results of deVV5 regions greater than 1 kb with 100% identity are linked by color-coded ribbons to the corresponding hit. (**b**) deVV5 genome annotated with results from global pairwise alignments. Each annotation corresponds to the longest region with 100% identity to the corresponding parental genome. deVV5 core and single ITR region are also displayed.

DNA from deVV5 and four parental virus strains was purified and sequenced by Next Generation Sequencing. The use of paired-end short reads and whole genome de novo assembly led to the generation of viral genomes with the core region and one copy of the Inverted Terminal Repeats (ITR) domain. These single-ITR versions of the genomes result from the use of unique k-mers in the contigs assembly process, but the presence of both ITR regions in every viral genome was confirmed by the mean depth coverage which was higher than the one from the core region, with a 2-fold factor (data not shown). Single-ITR versions of the genomes were retained for the sake of clarity in the subsequent paragraph. The resulting genome sequences, MVA (163,668 bp), COP (175,766 bp), WR (181,350 bp), WY (182,933 bp) and deVV5 (172,732 bp) are shown in Figure 3a around the circos plot. The links between the genomes reflect identical DNA sequences longer than 1000 bp between the deVV5 sequence and each of the four parental genomes. Some regions of deVV5 seem to be conserved in several parental strains but long regions (including ITRs) appeared to be specific to only one parental genome. Nevertheless, almost all the deVV5 genome was found to be 100% identical in at least one parental genome. To further investigate the composition of the deVV5 genome, global pairwise alignments were performed to identify the longest identical DNA regions between deVV5 and any parental genome (Figure 3b). 

Starting at the 5′ end of the core region, the first 30 kilobases of deVV5 are almost identical to WY sequence, followed by MVA (42 kb). The middle of the core region appears to be a patchwork of regions around 5 kb derived from the four strains. The 3′ end of the core domain is mainly composed of sequences identical to COP (56 kb). Finally, as described above, the 17-kb ITR domain is identical to WY. These observations confirmed that the shuffling resulted in an assembly of three main regions, derived from WY, MVA and COP for the core domain, surrounded by ITR regions derived from WY.

### 3.4. Arming of deVV5 Leads to Increased Potency 

**Figure 4:**
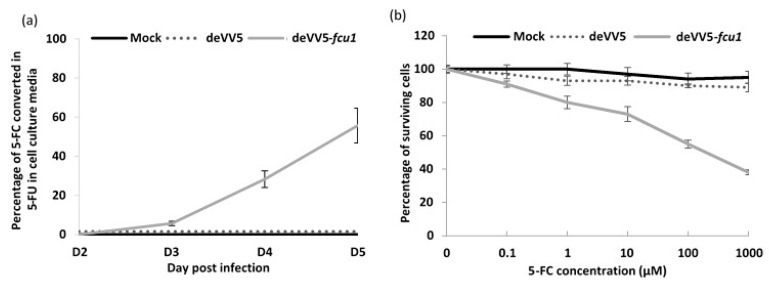
Functionality and efficacy of FCU1 expressed by deVV5. (**a**) Conversion of 5-fluorocytosine (5-FC) to 5-fluorouracil (5-FU) and release of 5-FU in the cell culture supernatant. HCT 116 cells were infected with the indicated vector at a MOI of 10^–5^ and then incubated with 0.1 mM 5-FC from day 2 to day 5 post infection. The relative concentration of 5-FC and 5-FU in the culture supernatant was measured by HPLC. The results are expressed as the percentage of 5-FU released relative to the total amount of 5-FC + 5-FU. Each data point represents the mean of triplicate determinations ± SD. (**b**) In vitro sensitivities of infected human tumor cells to 5-FC. HCT 116 human tumor cells were mock-infected or infected with deVV5 and deVV5-*fcu1* at a MOI of 10^–5^. After 48 h, cells were treated by increasing concentrations of 5-FC. Cell survival was determined 3 days later as described in Materials and Methods section. Cell viability results are expressed as the percentage of viable cells in the presence and absence of the prodrug. Values are represented as mean ± SD of triplicate determinations.

deVV5 was armed with a therapeutic gene, *FCU1* [28], inserted in the *TK* locus under the control of the strong p11k7.5 VACV promoter, to generate the deVV5-*fcu1* variant. The suicide gene *FCU1* encodes a protein that catalyzes the conversion of the non-toxic prodrug 5-FC into 5-FU and its derivatives toxic metabolites. Expression of a functional FCU1 protein by deVV5-*fcu1* was confirmed by quantification of 5-FU released into the supernatant of infected cells. The analysis of HCT 116 cells supernatants by HPLC showed a progressive release of 5-FU into the extracellular medium of HCT 116 cells infected with the indicated viruses at a MOI of 10^–5^ and incubated with 0.1 mM 5-FC (Figure 4a). Five days after infection, approximately 60% of 5-FC was converted into 5-FU into the supernatant of deVV5-*fcu1* infected cells. To compare the antitumoral activity of deVV5-*fcu1* vector alone or combined with 5-FC treatment, the HCT 116 human tumor cell line highly sensitive to 5-FU (data not shown) was infected at a MOI of 10^–5^ and 5-FC was added to the culture supernatants at a range of concentrations (10^–3^ to 10^–7^ M). Cell viability was determined five days post infection by trypan blue exclusion (Figure 4b). The addition of 5-FC had no impact on the viability of mock or deVV5 infected tumor cells. In contrast, the 5-FC conferred increased cytotoxicity in a prodrug dose-dependent manner to deVV5-*fcu1* infected human tumor cells. The combination of a low amount of deVV5-*fcu1* with 1 mM of 5-FC induced 60% mortality of HCT 116 cells. These results indicate that recombinant deVV5-*fcu1*, in the presence of the 5-FC prodrug, had acquired an enhanced in vitro anti-tumor activity as compared to deVV5 alone. Together, these in vitro enzymatic activity results demonstrate that deVV5-*fcu1* can express a functional therapeutic *FCU1* gene and is an efficient vector for viral directed enzyme prodrug therapy (VDEPT) *in vitro.*

### 3.5. Increased Oncolytic and Replicative Efficiency of deVV5 and deVV5-fcu1 

**Figure 5:**
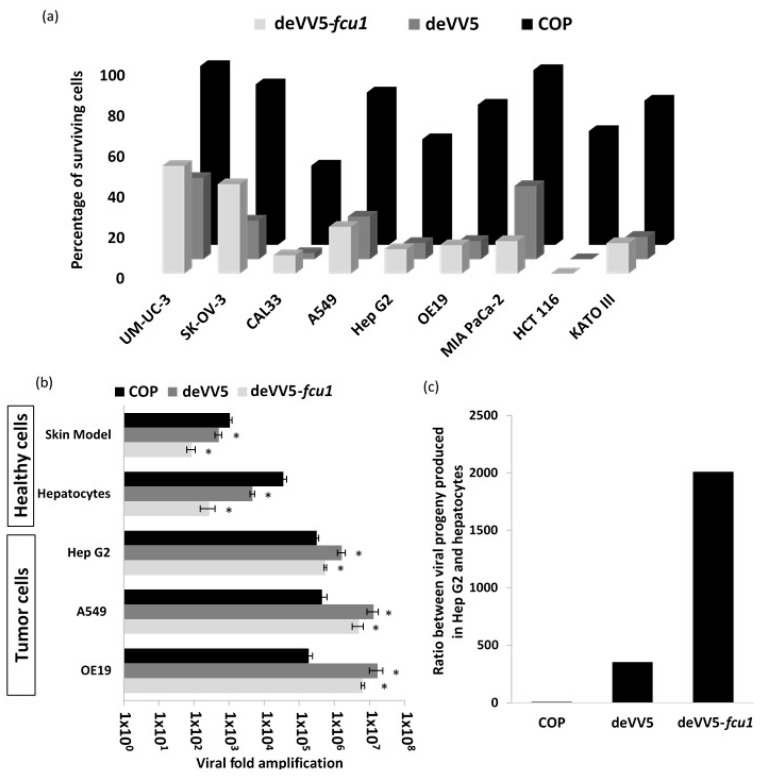
Both chimeric and recombinant chimera are able to infect and kill cancer cells leaving primary cells intact. (**a**) Oncolytic activities of COP, deVV5 and deVV5-*fcu1* by measuring the cell viability 5 days after infection of different cancer cell lines. Tumor cells were infected with the indicated viruses and cell viability was determined as described in the Materials and Methods section. The MOI used for each cell line was adjusted according to its susceptibility to COP infection. The MOI used for the infection was MOI 10^–5^ (3 PFU/well) for UM-UC-3, SK-OV-3, CAL33, A549 and Hep G2 cells, MOI 10^–4^ (30 PFU/well) for OE19, MIA PaCa-2 and HCT 116 cells and MOI 10^–3^ (300 PFU/well) for KATO III cells. (**b**) Replication in tumor cell lines and in primary human cells. Tumor cells were infected at MOI 10^–5^ and harvested 3 days post infection. Human primary hepatocytes were infected with 100 PFU/well and harvested 3 days post infection. 3D Phenion FT skin models were infected with 1.10^5^ PFU and harvested 7 days post infection. Viral progeny production was determined by plaque titration. Results are expressed as viral fold amplification (corresponding to output/input ratio). Asterisks denote statistical significance compared to COP (*p* < 0.05). (**c**) Ratio between viral production obtained in Hep G2 hepatocarcinoma cells and hepatocytes for the three viruses: COP, deVV5 and deVV5-*fcu1*.

To determine whether the armed and wild type evolved viruses can replicate and kill cancer cells, a screen of tumor cells from various origins was undertaken (Figure 5a). At low MOI, all tested cancer cells were more susceptible to the chimeric virus deVV5. Numerous cancer cell lines (UM-UC-3, HCT 116, A549 and SK-OV-3) were sensitive to deVV5 infection while remaining resistant to oncolysis by COP. These results were correlated with a significant increase in deVV5 replication in all tumor cell lines tested (Figure 5b). 

Compared to parental COP, deVV5 produced five times more viral particles in Hep G2 cells, 30 times more in A549 cells and 90 times more in OE19 cells. The deletion of the TK gene in deVV5 had a slight impact on the virus capacity to kill cancer cells. In most of the tested cell lines, deVV5-fcu1 demonstrated similar lytic activity (Figure 5a) and equivalent replication efficiency (Figure 5b) than the parental deVV5. These results confirmed the oncolytic benefit obtained through the process of directed evolution resulting in genomic blending and the possibility to delete genes without major impact on lytic and replication features of the virus in tumor cells.

### 3.6. Replication of deVV5 and deVV5-fcu1 on Human Primary Cells

Human hepatocytes and human reconstituted skin (Phenion full-thickness skin model) were used as primary healthy cells to evaluate the in vitro growth profiles of the newly generated viruses. As compared to COP, the chimeric wild type deVV5 demonstrated a reduced replication on primary cells (Figure 5b). A 2-fold and 10-fold reduction of replication were observed respectively in Phenion full-thickness skin model and hepatocytes. Moreover, TK deletion had a clear benefit on deVV5-*fcu1* allowing a supplementary 10-fold reduction of replication on human skin and hepatocytes. These results confirmed, and are consistent with previously reports demonstrating, the benefit of deleting *TK* gene for reducing virus replication in non-cancerous cells [34]. As a consequence, the ratio of viral progeny produced on Hep G2 tumor cells to that in human primary hepatocytes, was largely improved for the deVV5-*fcu1* where the transgene was inserted into the TK locus (Figure 5c). Taken together, these results demonstrate the benefit of the recombinant deVV5. 

## 4. Discussion

Directed evolution of therapeutic strains of oncolytic viruses is a powerful approach to isolate novel variants with desirable properties. We were able to isolate a new chimeric oncolytic virus, deVV5, by shuffling four VACV genomes, followed by selection under stringent conditions, allowing enrichment of candidates with increased oncolytic potency in vitro. deVV5 displays significant improved oncolytic potency in vitro compared to COP which had shown the highest oncolytic activity of the parental strains. To limit off-target replication and to evaluate its capacity to carry and express foreign therapeutic genes, deVV5 was modified by insertion of the *FCU1* gene into the *TK* locus. Both wild type chimeric virus deVV5 and the *TK*-deleted-armed deVV5-*fcu1* did replicate in most tumor cell lines tested. At very low MOI, i.e., in the range of 10^–5^, the new variants were able to infect and kill human cancer cell lines that are usually resistant to COP at the same MOI, such as HCT 116 (colon), MIA PaCa-2 (pancreas), SK-OV-3 (ovarian), UM-UC-3 (bladder) and A549 (lung). 

The ratio of viral replication in tumor cells compared to that in primary cells, exceeded 400 for deVV5 (Figure 5c), and was further improved 6-fold after TK deletion in deVV5-*fcu1*. This ratio was improved both by increase of the replicative activity on tumor cell lines and by decrease of the replication on primary cells. Considering the selection process on tumor cells, such a reduction of viral replication in non-cancerous cells was not expected. It is not clear yet whether this might result from components of MVA which is the only non-replicative strain within the four parental viruses. Nevertheless, the increased attenuation in both human skin and hepatocyte cells combined with improved oncolytic potency in vitro suggests that the directed evolution approach is a potent tool to generate improved OVs for cancer treatment. 

In this study, we also demonstrated that deVV5 can be armed with a therapeutic transgene without jeopardizing the oncolytic potency and the safety. We and others already demonstrated the potential of the FCU1/5-FC enzyme-prodrug system using a variety of delivery systems including replication-defective viruses [25,28] and replication-selective oncolytic viruses [12,34,35,36,37,38,39]. Our studies with the novel deVV5 confirm these findings.

The sequencing of the new deVV5 revealed the presence of sequences from each of the four parental strains. The presence of each parental genome, at different proportions, highlights the high frequency of homologous recombination between VACV strains. In this work, we decided to report the longest regions of deVV5 with strict identity to parental genomes, likely expected to be the result of homologous recombination events. However, it is important to notice that this representation does not include some shorter regions also identical to other parental strains because VACV genomes share high similarity (at least 90% sequence identity). The three main regions are identical to WY, COP and MVA strains respectively, whereas DNA of WR strain was found at a lower level in the deVV5 genome. The presence of a large region of DNA of the non-propagative MVA, indicates that recombination events appeared quickly, and that chimeric viruses are formed at early stages of the directed evolution process. Further characterization of deVV5 transcriptome and proteome, coupled with comparative genomics approaches should provide more insights on the gain of function associated with the oncolytic phenotype of deVV5. 

Prior to testing this virus in the clinical setting, further pre-clinical studies in vivo will be necessary to confirm an advantageous safety profile and improved oncolytic potency. In addition, it will be essential to investigate in vivo the immune profile induced by deVV5 in the tumor microenvironment. 

## 5. Conclusions

In conclusion, our studies show that directed evolution by shuffling different vaccinia viruses can generate recombinant poxviruses which demonstrate superior oncolytic potency in tumor cells accompanied by increased attenuation in normal cells in vitro. This is a powerful approach and could be adapted to generate novel oncolytic viruses for the treatment of specific cancer indications.

## Figures and Tables

**Table 1:** Percentage of surviving cells infected by four strains of vaccinia virus (VACV) used in this study. **Various** human tumor cell lines were infected by the four parental VACV strains (MVA, WY, WR and COP) at Multiplicity of Infection (MOI) 10^–5^. Survival was determined 5 days later, as described in the Materials and Methods section. Cell viability results are expressed as the percentage of viable cells relative to non-infected cells. Values are expressed as mean ± SD of three individual infections.

Cell Lines	MVA	WY	WR	COP
A549	94.5 ± 3.6	90.2 ± 3.2	58.6 ± 3.7	47.8 ± 4.8
LoVo	98.6 ± 2.7	90.4 ± 5.9	70.8 ± 6.2	73.1 ± 5.2
MIA PaCa-2	103.2 ± 4.2	92.8 ± 2.9	89.4 ± 2.4	78.1 ± 1.5
U-87 MG	102.8 ± 4.8	64.6 ± 1.6	41.0 ± 0.8	43.6 ± 0.3
Hep G2	97.3 ± 3.9	66.8 ± 4.9	70.9 ± 9.4	13.6 ± 2.7
HCT 116	99.2 ± 5.4	91.5 ± 3.4	94.6 ± 3.4	55.1 ± 7.5
OE19	105.2 ± 6.2	78.9 ± 2.2	77.6 ± 3.3	70.3 ± 0.7
SK-OV-3	95.4 ± 7.3	59.8 ± 1.8	99.7 ± 1.5	42.9 ± 1.3
